# Supplementary material for: Mitochondrial protein biogenesis in the synapse is supported by local translation
Source: EMBO Rep. 2020 Jun 18;21(8):e48882. doi: 10.15252/embr.201948882 (PMC7403725; doi:10.15252/embr.201948882)
Supplement: Supplementary file 1 — Expanded View Figures PDF [file EMBR-21-e48882-s001.pdf]

Expanded View Figures

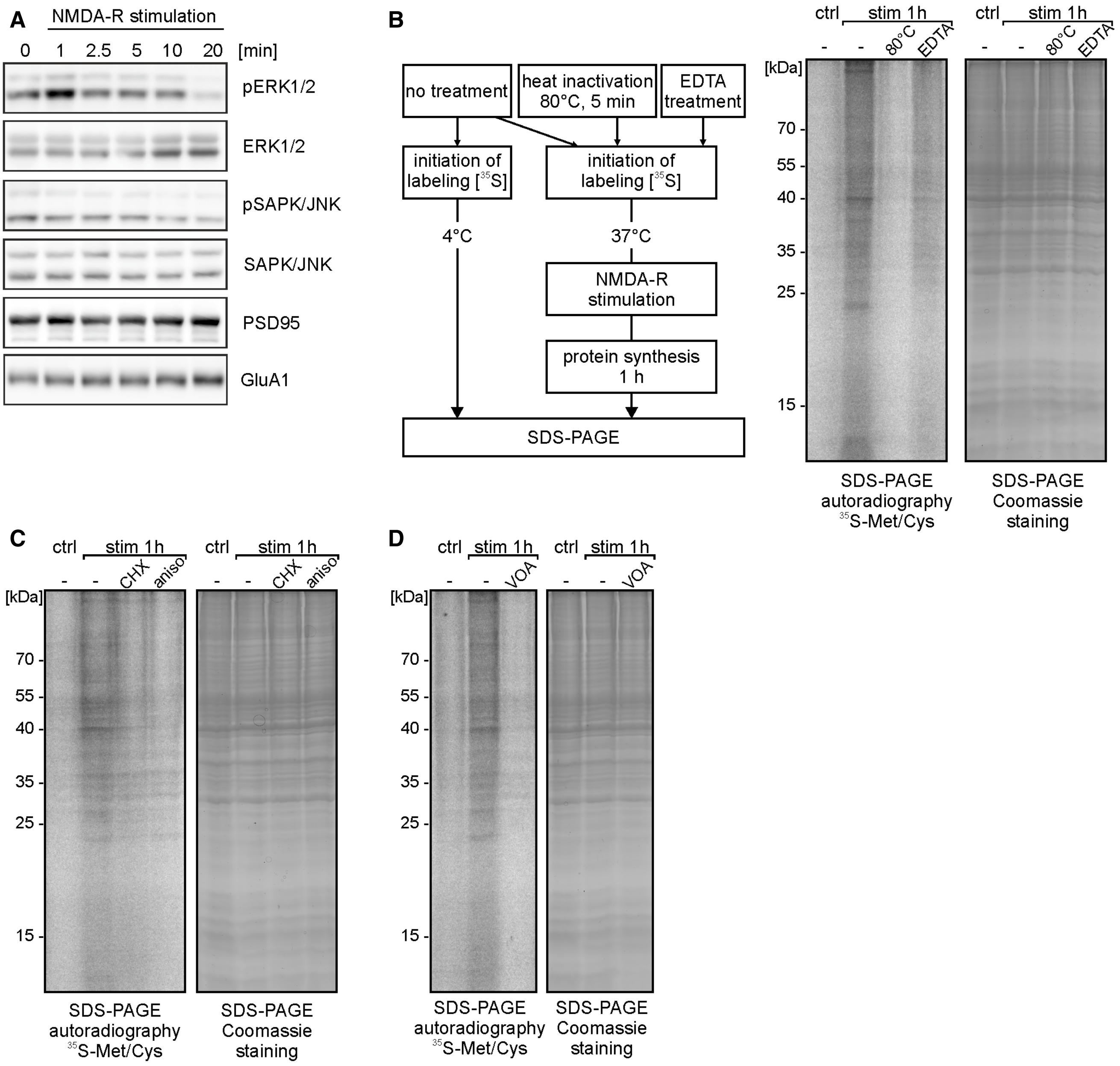

Figure EV1.

© 2020 The Authors

Figure EV1. Related to Fig 1. NMDA-R stimulation of synaptoneurosomes leads to transient ERK1/2 phosphorylation and induces *de novo* protein synthesis.

A Western blot on synaptoneurosomes verifying activation of selected kinases in response to the stimulation (0-20 min). Chosen NMDA-R stimulation protocol leads to transient phosphorylation of extracellular signal-regulated protein kinases 1 and 2 (ERK1/2). In contrast, stress-activated protein kinase/c-Jun NH2-terminal kinase (SAPK/JNK) is not phosphorylated upon treatment. Antibodies recognizing total ERK1/2 and total SAPK/JNK were used to ensure equal protein levels of analyzed kinases. Anti-PSD95 and anti-GluA1 antibodies were used to verify equal protein loading.

B Schematic representation of the control experiments (left panel). To rule out the possibility of stickiness of the labeled amino acids, SN were inactivated at 80°C for 5 min or pretreated with EDTA (50 mM) to disrupt polyribosomes. Next, synaptoneurosomes (untreated, heat-inactivated, and EDTA-treated) were NMDA-R-stimulated and incubated with radioactive ^{35}S-methionine/cysteine mix for 1 h. SDS–PAGE autoradiography shows newly synthesized proteins in SN, labeled with [^{35}S]. Heat inactivation of SN as well as EDTA treatment inhibited ^{35}S-methionine/cysteine incorporation into SN.

C, D Synaptoneurosomes were stimulated in the presence of protein synthesis inhibitors (cycloheximide, anisomycin) (C) or VOA mixture (containing 1 μM valinomycin, 20 μM oligomycin, 8 μM antimycin) (D). Inhibition of ^{35}S-methionine/cysteine incorporation into *de novo* synthetized proteins in synaptoneurosomes was observed.

Source data are available online for this figure.

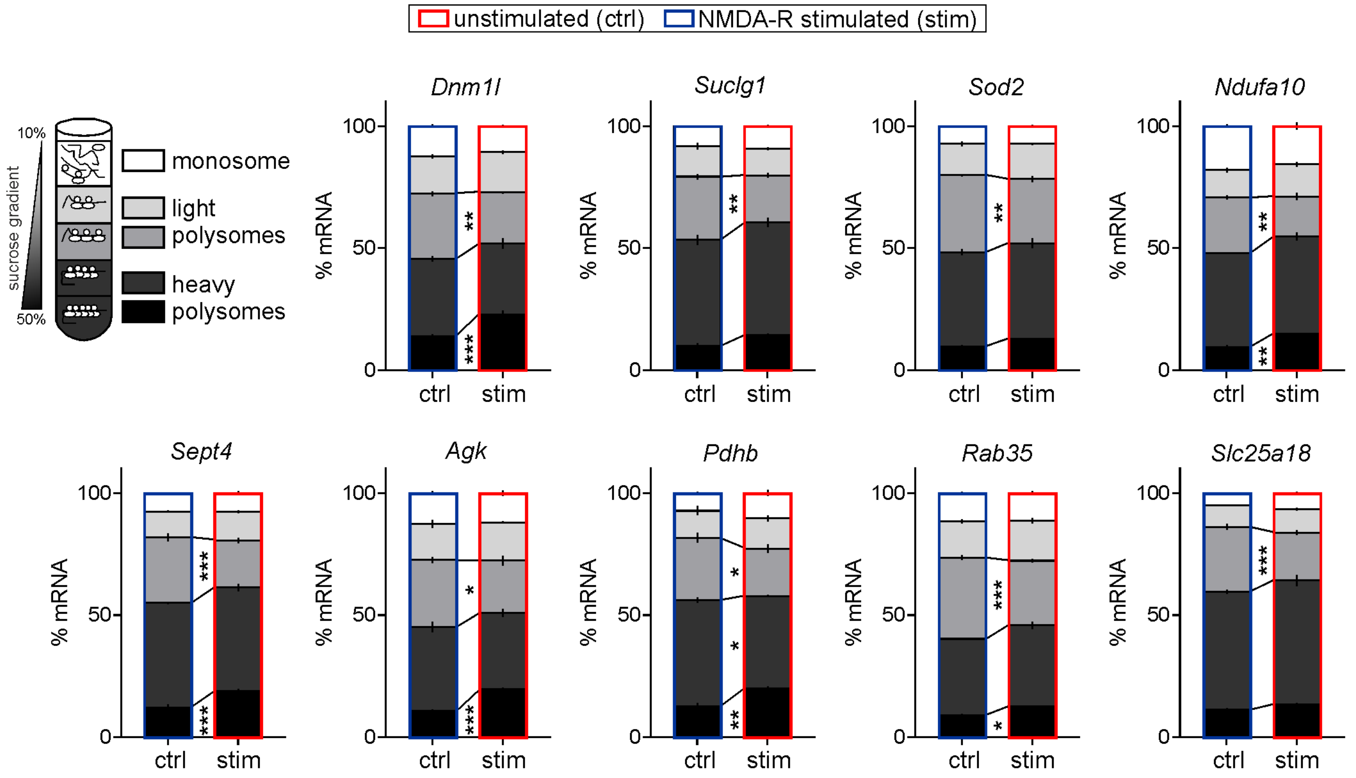

Figure EV2. Related to Fig 2. Validation of RNA-seq data using qRT–PCR on polysomal fractions.

Graphs show percentage of mRNAs in different fractions from unstimulated and NMDA-R-stimulated synaptoneurosomes (20 min stimulation). Fractions as in Fig 2: fraction I—monosome, fractions II-III—light polyribosomes, and fractions IV-V—heavy polyribosomes. Selected transcripts encoding for mitochondrial proteins shift toward the heavy polyribosomal fractions upon the NMDA-R stimulation (n = 4 biological replicates; *P < 0.05, **P < 0.01, ***P < 0.001; one-way ANOVA, *post hoc* Sidak's multiple comparisons test; error bars indicate SEM).

© 2020 The Authors

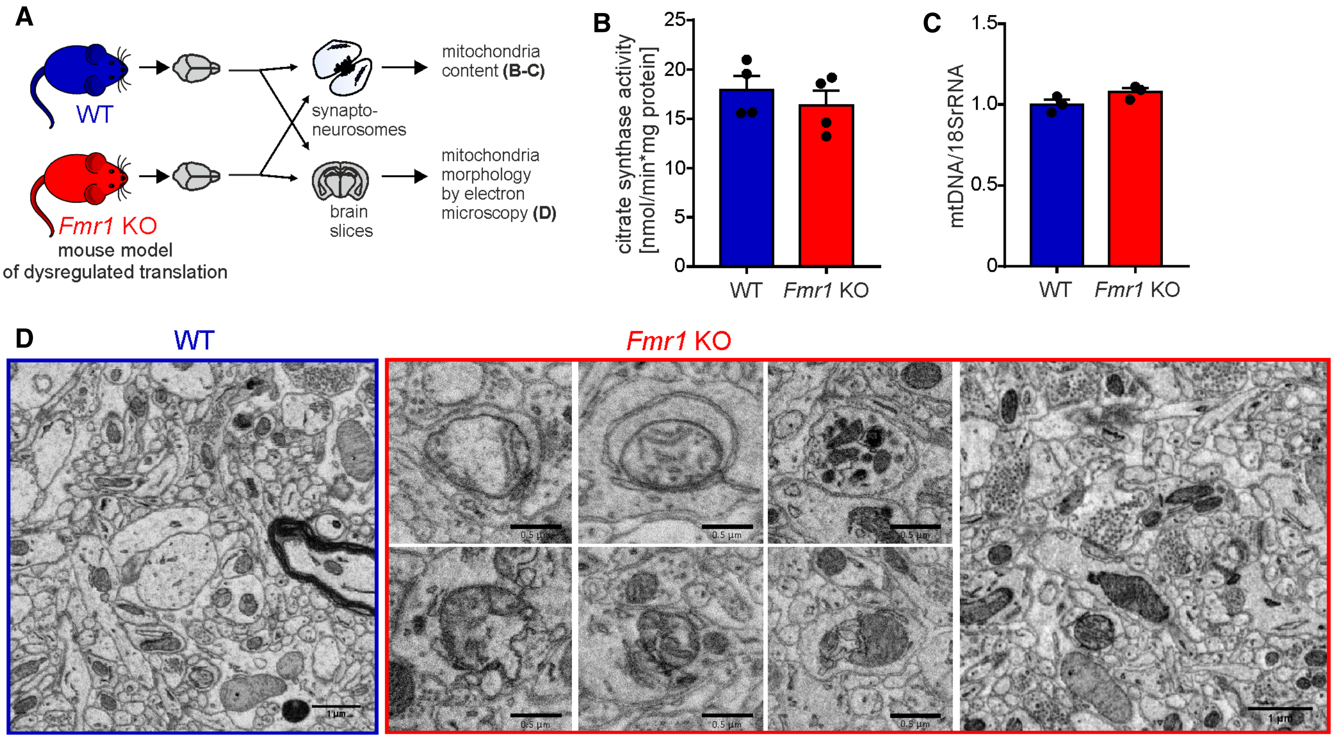

Figure EV3. Related to Fig 5. Altered mitochondrial ultrastructure but not mitochondria content in *Fmr1* KO synapses.

A *Fmr1* KO mice were used as a mouse model of dysregulated local translation. Mitochondria content in synaptoneurosomes and mitochondria morphology in the brain slices were assessed in *Fmr1* KO and WT mice.

B Measurements of enzymatic activity of citrate synthase in *Fmr1* KO and WT synaptoneurosomes did not reveal any differences between the two groups (n = 4 per genotype, P = 0.47; unpaired two-tailed t-test; error bars indicate SEM).

C Mitochondria content in *Fmr1* KO and WT synaptoneurosomes was determined based on qPCR analysis of mitochondrial DNA levels (MT-ND1) relative to 18S rRNA. No significant difference in mitochondrial number among the genotypes was detected (n = 3 per genotype; P = 0.1108; unpaired two-tailed t-test; error bars indicate SEM).

D Ultrastructure of WT and *Fmr1* KO brain sections assessed using electron microscopy. Images are presented in reverse contrast, with membranes being black. In the synapses of WT mice, we observed morphologically normal mitochondria with regular distribution of cristae and matrix of typical electron density. In contrast, striking morphological abnormalities were observed in *Fmr1* KO neurons. Scale bars, 1 µm (leftmost and rightmost images) and 0.5 µm (middle images).

 © 2020 The Authors
