# Supplementary material for: Mitochondrial protein biogenesis in the synapse is supported by local translation
Source: EMBO Rep. 2020 Jun 18;21(8):e48882. doi: 10.15252/embr.201948882 (PMC7403725; doi:10.15252/embr.201948882)
Supplement: Supplementary file 2 — Table EV1 [file EMBR-21-e48882-s002.docx]

| Table EV1 MS identification of proteins present in the bands 1-8 (Fig. 3D) | | | | | | | |
| --- | --- | --- | --- | --- | --- | --- | --- |
| Band 1 | **Band 2** | **Band 3** | **Band 4** | **Band 5** | **Band 6** | **Band 7** | **Band 8** |
| Atp5a1 | Abat | Atp5a1 | Atp5a1 | Atp5a1 | Cox4i1 | Apoo | Atp5a1 |
| Ckmt1 | Aco2 | Atp5b | Atp5b | Atp5b | Cox5a | Atp5a1 | Atp5b |
| Cox5a | Aldh2 | Atp5c1 | Atp5c1 | Atp5c1 | Cox5b | Atp5b | Atp5c1 |
| Pdha1 | Aldh4a1 | Atp5c1 | Atp5d | Atp5d | Cox6b1 | Atp5c1 | Atp5d |
| Pdhb | Aldh5a1 | Atp5d | Atp5e | Atp5e | Cox6c | Atp5d | Atp5e |
| Prdx2 | Aldh6a1 | Atp5e | Atp5f1 | Atp5f1 | Cox7a2 | Atp5e | Atp5f1 |
| Prdx5 | Atp5a1 | Atp5f1 | Atp5o | Atp5o | Cox7a2l | Atp5f1 | Atp5j2 |
| Tkt | Atp5b | Atp5j | Ndufa10 | Ndufa10 | Cyc1 | Atp5j2 | Atp5l |
| Tpi1 | Cat | Atp5j2 | Ndufa11 | Ndufa11 | Dbt | Atp5l | Atp5o |
| Tubb3 | Ckmt1 | Atp5l | Ndufa12 | Ndufa12 | Ndufa1 | Atp5o | Cox4i1 |
| Vdac1 | Cox4i1 | Atp5o | Ndufa13 | Ndufa13 | Ndufa10 | Chchd3 | Cox5a |
|  | Cox5a | Ckmt1 | Ndufa2 | Ndufa2 | Ndufa11 | Ckmt1 | Cox5b |
|  | Cox5b |  | Ndufa3 | Ndufa3 | Ndufa12 | Cox4i1 | Cox6b1 |
|  | Cox6c |  | Ndufa5 | Ndufa4 | Ndufa13 | Cox5a | Cox6c |
|  |  |  | Ndufa6 | Ndufa5 | Ndufa2 | Cox5b | Cox7a2 |
|  |  |  | Ndufa7 | Ndufa6 | Ndufa3 | Cox6b1 | Cox7a2l |
|  |  |  | Ndufa8 | Ndufa7 | Ndufa4 | Cox6c | Ndufa10 |
|  |  |  | Ndufa9 | Ndufa8 | Ndufa5 | Cox7a2 | Ndufa11 |
|  |  |  | Ndufab1 | Ndufa9 | Ndufa6 | Cox7a2l | Ndufa12 |
|  |  |  | Ndufb10 | Ndufab1 | Ndufa7 | Ndufa1 | Ndufa13 |
|  |  |  | Ndufb11 | Ndufb10 | Ndufa8 | Ndufa10 | Ndufa2 |
|  |  |  | Ndufb3 | Ndufb11 | Ndufa9 | Ndufa11 | Ndufa3 |
|  |  |  | Ndufb4 | Ndufb3 | Ndufab1 | Ndufa12 | Ndufa4 |
|  |  |  | Ndufb5 | Ndufb4 | Ndufb10 | Ndufa13 | Ndufa5 |
|  |  |  | Ndufb6 | Ndufb5 | Ndufb11 | Ndufa2 | Ndufa6 |
|  |  |  | Ndufb7 | Ndufb6 | Ndufb3 | Ndufa3 | Ndufa7 |
|  |  |  | Ndufb8 | Ndufb7 | Ndufb4 | Ndufa4 | Ndufa8 |
|  |  |  | Ndufc2 | Ndufb8 | Ndufb5 | Ndufa5 | Ndufa9 |
|  |  |  | Ndufs1 | Ndufb9 | Ndufb6 | Ndufa6 | Ndufb10 |
|  |  |  | Ndufs2 | Ndufc2 | Ndufb7 | Ndufa7 | Ndufb11 |
|  |  |  | Ndufs3 | Ndufs1 | Ndufb8 | Ndufa8 | Ndufb3 |
|  |  |  | Ndufs4 | Ndufs1 | Ndufb9 | Ndufa9 | Ndufb4 |
|  |  |  | Ndufs5 | Ndufs2 | Ndufc2 | Ndufab1 | Ndufb5 |
|  |  |  | Ndufs6 | Ndufs3 | Ndufs1 | Ndufb10 | Ndufb6 |
|  |  |  | Ndufs7 | Ndufs4 | Ndufs2 | Ndufb11 | Ndufb7 |
|  |  |  | Ndufs8 | Ndufs5 | Ndufs3 | Ndufb3 | Ndufb8 |
|  |  |  | Ndufv1 | Ndufs6 | Ndufs4 | Ndufb4 | Ndufb9 |
|  |  |  | Ndufv2 | Ndufs7 | Ndufs5 | Ndufb5 | Ndufc2 |
|  |  |  | Ndufv3 | Ndufs8 | Ndufs6 | Ndufb6 | Ndufs1 |
|  |  |  |  | Ndufv1 | Ndufs7 | Ndufb7 | Ndufs1 |
|  |  |  |  | Ndufv2 | Ndufs8 | Ndufb8 | Ndufs2 |
|  |  |  |  | Ndufv3 | Ndufv1 | Ndufb9 | Ndufs3 |
|  |  |  |  | Ndufv3 | Ndufv2 | Ndufc2 | Ndufs4 |
|  |  |  |  | Uqcr10 | Ndufv3 | Ndufs1 | Ndufs5 |
|  |  |  |  | Uqcrb | Ndufv3 | Ndufs1 | Ndufs6 |
|  |  |  |  | Uqcrc1 | Uqcr10 | Ndufs2 | Ndufs7 |
|  |  |  |  | Uqcrc2 | Uqcrb | Ndufs3 | Ndufs8 |
|  |  |  |  | Uqcrfs1 | Uqcrc1 | Ndufs4 | Ndufv1 |
|  |  |  |  | Uqcrh | Uqcrc2 | Ndufs5 | Ndufv2 |
|  |  |  |  | Uqcrq | Uqcrfs1 | Ndufs6 | Ndufv3 |
|  |  |  |  |  | Uqcrh | Ndufs7 | Ndufv3 |
|  |  |  |  |  | Uqcrq | Ndufs8 | Uqcr10 |
|  |  |  |  |  |  | Ndufv1 | Uqcrb |
|  |  |  |  |  |  | Ndufv2 | Uqcrc1 |
|  |  |  |  |  |  | Ndufv3 | Uqcrc2 |
|  |  |  |  |  |  | Ndufv3 | Uqcrfs1 |
|  |  |  |  |  |  | Uqcr10 | Uqcrh |
|  |  |  |  |  |  | Uqcrb | Uqcrq |
|  |  |  |  |  |  | Uqcrc1 | Vdac1 |
|  |  |  |  |  |  | Uqcrc2 | Vdac1 |
|  |  |  |  |  |  | Uqcrfs1 | Vdac2 |
|  |  |  |  |  |  | Uqcrh | Vdac3 |
|  |  |  |  |  |  | Uqcrq |  |
|  |  |  |  |  |  | Vdac1 |  |
|  |  |  |  |  |  | Vdac2 |  |
|  |  |  |  |  |  | Vdac3 |  |
