# Supplementary material for: Mitochondrial protein biogenesis in the synapse is supported by local translation
Source: EMBO Rep. 2020 Jun 18;21(8):e48882. doi: 10.15252/embr.201948882 (PMC7403725; doi:10.15252/embr.201948882)
Supplement: Supplementary file 3 — Table EV2 [file EMBR-21-e48882-s003.docx]

**Table EV2: KEY RESOURCES**

| REAGENT or RESOURCE | SOURCE | IDENTIFIER |  |
| --- | --- | --- | --- |
| Antibodies | | | |
| mouse monoclonal anti-PSD95 | Merck Millipore | Cat#MAB1598 |  |
| rabbit polyclonal anti-GluA1 | Thermo Fisher Scientific | Cat#PA1-37776 |  |
| rabbit monoclonal anti-GluA2 | Cell Signaling | Cat#13607 |  |
| rabbit polyclonal anti-Nlgn3 | Synaptic Systems | Cat#129113 |  |
| mouse monoclonal anti-synaptophysin | Merck Millipore | Cat#MAB329 |  |
| mouse monoclonal anti-α-tubulin | Santa Cruz Biotechnology | Cat# sc-134239 |  |
| rabbit polyclonal anti-GFAP | Proteintech | Cat#16825-1-AP |  |
| mouse monoclonal anti-GAPDH | Merck Millipore | Cat#MAB374 |  |
| rabbit polyclonal anti-Hsp90 | Abcam | Cat#ab13495 |  |
| rabbit monoclonal anti-KDM1/LSD1 | Abcam | Cat#ab129195 |  |
| rabbit monoclonal anti-cJun | Cell Signaling | Cat#9165 |  |
| MAPK Family Antibody Sampler Kit | Cell Signaling | Cat#9926 |  |
| Phospho-MAPK Family Antibody Sampler Kit | Cell Signaling | Cat#9910 |  |
| peroxidase anti-mouse IgG (H+L) | Vector Laboratories | Cat#PI-2000 |  |
| peroxidase anti-rabbit IgG (H+L) | Vector Laboratories | Cat#PI-1000 |  |
| custom rabbit polyclonal anti-Ndufb8 | Peter Rehling Lab | PRAB3765 |  |
| mouse monoclonal anti-SDHA (D-4) | Santa Cruz Biotechnology | Cat#sc-166947 |  |
| mouse monoclonal anti-Uqcrc2 | Abcam | Cat#ab14745 |  |
| custom rabbit polyclonal anti-COX6A | Peter Rehling Lab | PRAB3283 |  |
| mouse monoclonal anti-Atp5a antibody [15H4C4] | Abcam | Cat#ab14748 |  |
| Bacterial and Virus Strains | | | |
|  |  |  |  |
| Biological Samples |  |  |  |
|  |  |  |  |
| Chemicals, Peptides, and Recombinant Proteins | | | |
| cOmplete™, EDTA-free Protease Inhibitor Cocktail | Roche | Cat#05056489001 |  |
| RiboLock RNase Inhibitor | Thermo Fisher Scientific | Cat#EO0381 |  |
| N-Methyl-D-aspartic acid (NMDA) | Sigma-Aldrich | Cat#M3262 |  |
| L-Glutamic acid monosodium salt hydrate | Sigma-Aldrich | Cat#G5889 |  |
| DL-2-Amino-5-phosphonovaleric Acid (APV) | Sigma-Aldrich | Cat#A5282 |  |
| cycloheximide | Sigma-Aldrich | Cat#C1988 |  |
| puromycin dihydrochloride from *Streptomyces alboniger* | Sigma-Aldrich | Cat#P8833 |  |
| chloramphenicol | Sigma-Aldrich | Cat#C0378 |  |
| digitonin | Calbiochem | Cat#300410 |  |
| ^35^S-Met/Cys methionine/cysteine mix | Perkin Elmer | Cat#NEG072002MC |  |
| HMW Native Marker Kit | GE Healthcare | Cat#17044501 |  |
| TRIzol™ Reagent | Thermo Fisher Scientific | Cat#15596026 |  |
| TURBO DNase | Ambion | Cat#AM2238 |  |
| dNTP Set | Thermo Fisher Scientific | Cat#R0182 |  |
| dUTP Solution | Thermo Fisher Scientific | Cat# R0133 |  |
| E. coli DNA Ligase | NEB | Cat#M0205L |  |
| E. coli DNA Polymerase I | NEB | Cat#M0209L |  |
| RNase H | Thermo Fisher Scientific | Cat#18021071 |  |
| USER Enzyme | NEB | Cat#M5505L |  |
| UDG Reaction Buffer | NEB | Cat#M0280S |  |
| Phusion High-Fidelity DNA Polymerase | Thermo Fisher Scientific | Cat#F530L |  |
| iTRAQ Reagent - 8Plex 25 U bulk pack | Sciex | Cat#4393528 |  |
| TMT10-plex isobaric label reagent set | Thermo Fischer Scientific | Cat#90111 |  |
| Trypsin (mass spectrometry) | Promega | Cat#V5280 |  |
| 1M Triethylammonium bicarbonate (TEAB) | Thermo Fischer Scientific | Cat#90114 |  |
| MMTS, methyl methanethiosulfonate | Pierce (Thermo Fischer) | Cat#23011 |  |
| IAA, iodoacetamide | BioUltra (Sigma) | Cat#I1149-5G |  |
| Chloroform | POCH | Cat#234431116 |  |
| Methanol | POCH | Cat#621990110 |  |
| 50% Hydroxylamine | Pierce (Thermo Fischer) | Cat#90115 |  |
| Acetonitrile | J.T.Baker | Cat#9821 |  |
| Glutaraldehyde 25% EM Grade 10 x 10ml ampoule | Agar scientific | Cat#AGR1020 |  |
| Potassium hexacyanoferrate(II) | Chempur | Cat#117469804 |  |
| Osmium Tetroxide 4% Solution (Pack of 5 x 2ml ampoules) | Agar scientific | Cat#AGR1023 |  |
| Thiocarbohydrazide 1g | Agar scientific | Cat#AGR1240 |  |
| MF-Millipore™ Membrane Filter, 0.22 µm pore size | MERCK | Cat#GSWP04700 |  |
| L-Aspartic Acid | Sigma | Cat#A9256 |  |
| Lead Nitrate | Agar scientific | Cat#AGR1217 |  |
| Durcupan™ ACM | MERCK (Sigma-Aldrich) | Cat#44610-1EA |  |
| ACLAR® Embedding Film, 7.8 mil thick, 8 x 12.5" (203 x 318mm) | TED PELLA | Cat#10501-10 |  |
| paraformaldehyde solution | Chempur | Cat#114321734 |  |
| EGTA | Sigma | Cat#E4378 |  |
| Lactobionic acid | Sigma | Cat#153516 |  |
| Taurine | Sigma | Cat#T0625 |  |
| HEPES | Sigma | Cat#H3375 |  |
| D-sucrose | Roth | Cat#4621.3 |  |
| BSA | Sigma | Cat#A6003 |  |
| CCCP | Sigma | Cat#C2759 |  |
| oligomycin | Sigma | Cat#O4876 |  |
| malic acid | Sigma | Cat#M0875 |  |
| sodium pyruvate | Sigma | Cat#P3280 |  |
| glutamic acid | Sigma | Cat#G1251 |  |
| rotenone | Sigma | Cat#R8875 |  |
| succinic acid | Sigma | Cat#S9512 |  |
| malonic acid | Sigma | Cat#M1296 |  |
| ascorbic acid | Sigma | Cat#A0278 |  |
| TMPD | Sigma | Cat#T3134 |  |
| sodium azide | Sigma | Cat#S2002 |  |
| glycerol | Roth | Cat#3783.2 |  |
| Triton X-100 | Sigma | Cat#X100 |  |
| acetyl-coenzyme A | Sigma | Cat#A2181 |  |
| DTNB | Sigma | Cat#D8130 |  |
| oxaloacetate | Sigma | Cat#O4126 |  |
|  |  |  |  |
| Critical Commercial Assays | | | |
| Pierce BCA protein assay kit | Thermo Fisher Scientific | Cat#23225 |  |
| TGX Stain-Free FastCast Acrylamide Kit, 10% | BioRad | Cat#161-0183 |  |
| TGX Stain-Free FastCast Acrylamide Kit, 12% | BioRad | Cat#161-0185 |  |
| Amersham ECL Prime Western Blotting Detection Reagent | GE Healthcare | Cat#RPN2236 |  |
| Ribo-Zero Gold rRNA-removal kit H/R/M | Illumina | Cat#MRZG12324 |  |
| SuperScript III Reverse Transcriptase | Thermo Fisher Scientific | Cat#18080085 |  |
| Second Strand Synthesis Buffer | Thermo Fisher Scientific | Cat#10812014 |  |
| High Sensitivity DNA kit | Agilent | Cat#5067-4626 |  |
| KAPA Universal Library Quantification Kit | Kapa Biosystems | Cat#KK4824 |  |
| SuperScript IV First-Strand Synthesis System | Thermo Fisher Scientific | Cat#18091050 |  |
| LightCycler® 480 Probes Master | Roche | Cat#04887301001 |  |
| TaqMan Gene Expression Assays: | Thermo Fisher Scientific | Cat# 4331182;  Assay ID: |  |
| TaqMan Gene Expression Assay Dnm1l |  | Mm01342903_m1 |  |
| TaqMan Gene Expression Assay Suclg1 |  | Mm00451244_m1 |  |
| TaqMan Gene Expression Assay Sod2 |  | Mm01313000_m1 |  |
| TaqMan Gene Expression Assay Ndufa10 |  | Mm00600325_m1 |  |
| TaqMan Gene Expression Assay Sept4 |  | Mm00448225_m1 |  |
| TaqMan Gene Expression Assay Agk |  | Mm01282171_s1 |  |
| TaqMan Gene Expression Assay Pdhb |  | Mm00499323_m1 |  |
| TaqMan Gene Expression Assay Rab35 |  | Mm01204416_m1 |  |
| TaqMan Gene Expression Assay Slc25a18 |  | Mm01183193_m1 |  |
| TaqMan Gene Expression Assay AT3G14080 |  | At02174019_g1 |  |
| Direct Detect | Merck | Cat#DDHW00010-WW |  |
| MitoPlate S-1 | Biolog | Cat#14105 |  |
| Biolog MAS | Biolog | Cat#72303 |  |
| Biolog Redox Dye Mix MC | Biolog | Cat#74353 |  |
| Deposited Data | | | |
| RNA-Sequencing data | This paper | <https://www.ncbi.nlm.nih.gov/geo/query/acc.cgi?acc=GSE122724> |  |
| Proteomic data | This paper | PRIDE archive px-submissions PXD012746 and PXD012707 |  |
| Experimental Models: Cell Lines | | | |
| HEK293T cells | ATCC |  |  |
| Experimental Models: Organisms/Strains | | | |
| Mouse: FVB/NJ | Jackson Laboratories | Stock No.: 001800 |  |
| Oligonucleotides | | | |
| random primers | GeneON | Cat#S300 |  |
| ERCC RNA Spike-In Control Mix 1 | Ambion | Cat#4456740 |  |
| MT-Nd1 fwd qRTPCR primer: CACTCCTCGTCCCCATTCTA | This paper | N/A |  |
| MT-Nd1 rev qRTPCR primer: ATGCCGTATGGACCAACAAT | This paper | N/A |  |
| 18s rRNA fwd qRTPCR primer: CGCGGTTCTATTTTGTTGGT | Kalita et. al, 2006 | N/A |  |
| 18s rRNA rev qRTPCR primer: AGTCGGCATCGTTTATGGTC | Kalita et. al, 2006 | N/A |  |
| Recombinant DNA | | | |
|  |  |  |  |
| Software and Algorithms | | | |
| GraphPad Prism 7.0 | GraphPad | https://www.graphpad.com/scientific-software/prism/ |  |
| Max-Quant | MaxQuant and Perseus are developed by the Computational Systems Biochemistry under Prof. Jürgen Cox. | 1.5.6.5 and 1.5.7.4 |  |
| Perseus |  | 1.6.0.7 |  |
| Scaffold 4 Q+S | Proteome Software |  |  |
| Cytoscape | Cytoscape Consortium | 3.6.0 |  |
| HTseq, RSeQC, BEDtools and SAMtools packages | Anders et al., 2015; Li et al., 2009; Quinlan and Hall, 2010; Wang et al., 2016 |  |  |
| STAR | Dobin et al., 2013 | https://github.com/alexdobin/STAR |  |
| DESeq2 Bioconductor R package | Love et al., 2014 |  |  |
| ImageQuant software | GE Healthcare |  |  |
| DatLab 7 | Oroboros Instruments |  |  |
| Other | | | |
| Phase Lock Gel Heavy 2 ml Tubes | 5Prime | Cat#2302830 |  |
| Nylon Net Filters; 100 µm, 60 µm, 30 µm, 10 µm | Merck Millipore | Cat#: NY1H02500, NY6002500, NY3002500, NY1002500 |  |
