# Supplementary figures and images for: Mitochondrial protein biogenesis in the synapse is supported by local translation
Source: EMBO Rep. 2020 Jun 18;21(8):e48882. doi: 10.15252/embr.201948882 (PMC7403725; doi:10.15252/embr.201948882)

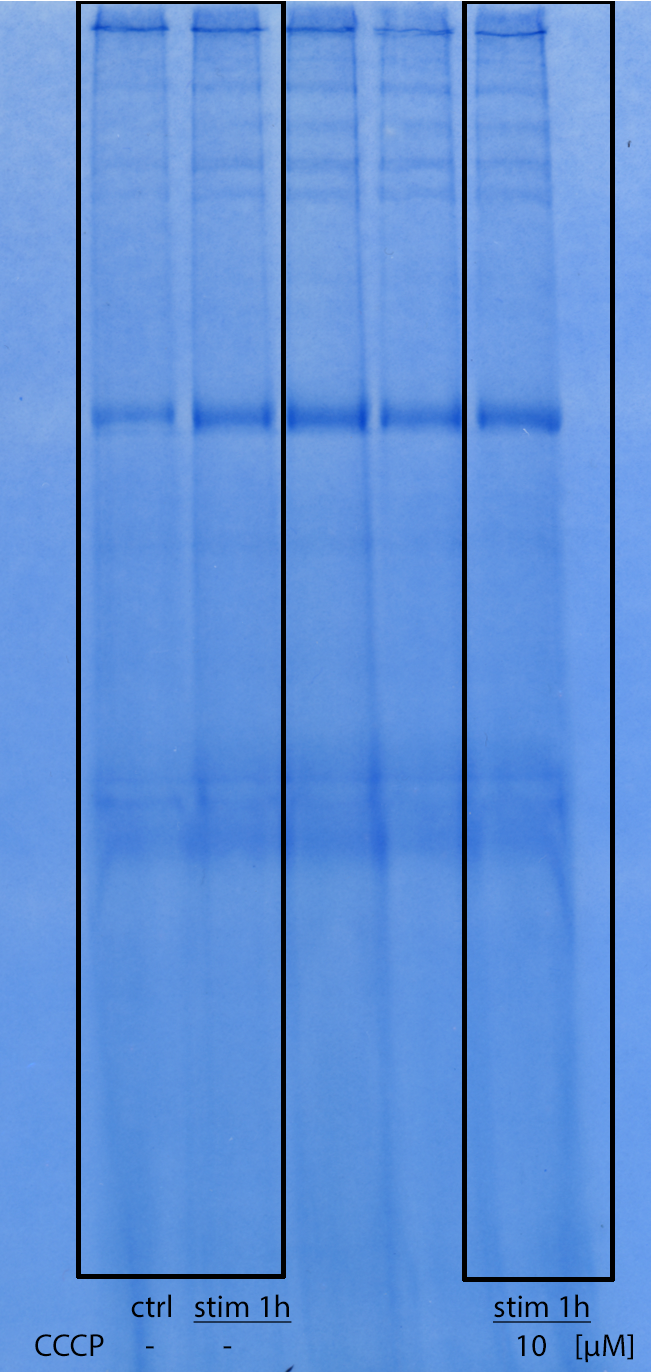

Supplement: Supplementary file 7 — Source Data for Figure 4 [file EMBR-21-e48882-s005.zip › Figure4_Source_Data/EMBOR-2019-48882V3-Figure_4B_Source_Data-sd(1).tif]

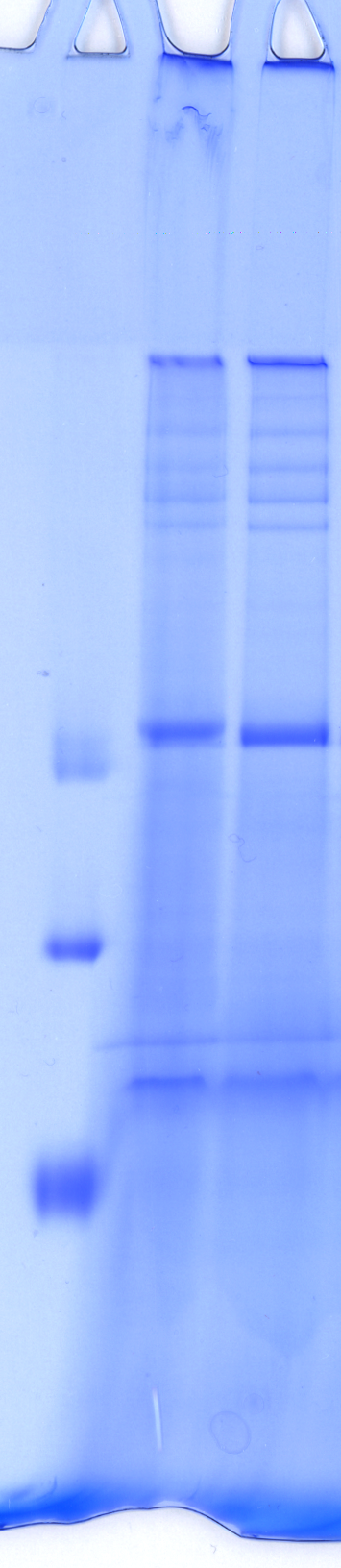

Supplement: Supplementary file 7 — Source Data for Figure 4 [file EMBR-21-e48882-s005.zip › Figure4_Source_Data/EMBOR-2019-48882V3-Figure_4A_Source_Data-sd.tif]

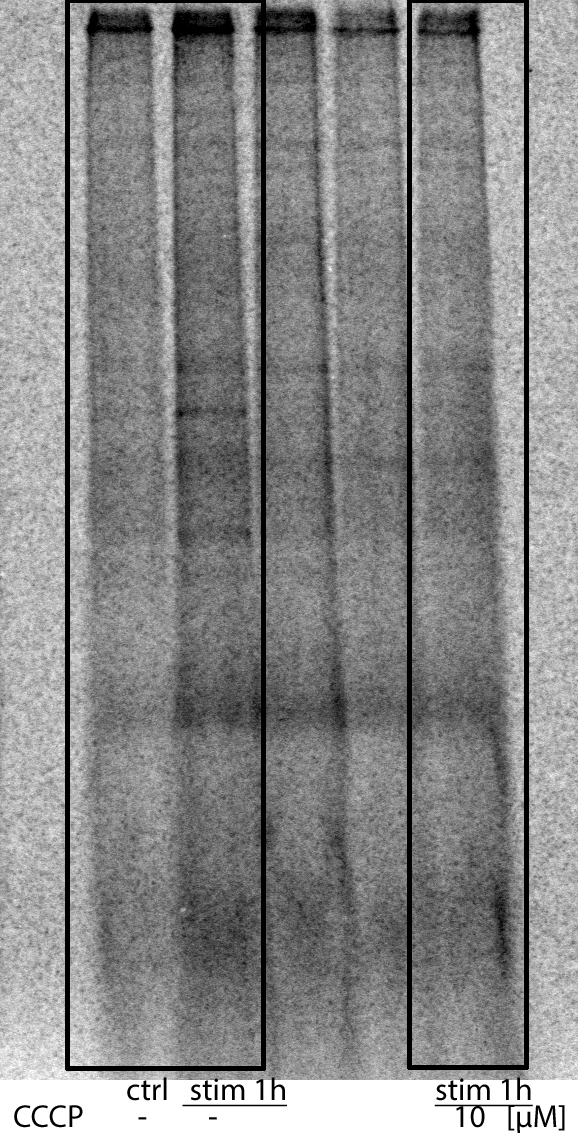

Supplement: Supplementary file 7 — Source Data for Figure 4 [file EMBR-21-e48882-s005.zip › Figure4_Source_Data/EMBOR-2019-48882V3-Figure_4B_Source_Data-sd.tif]

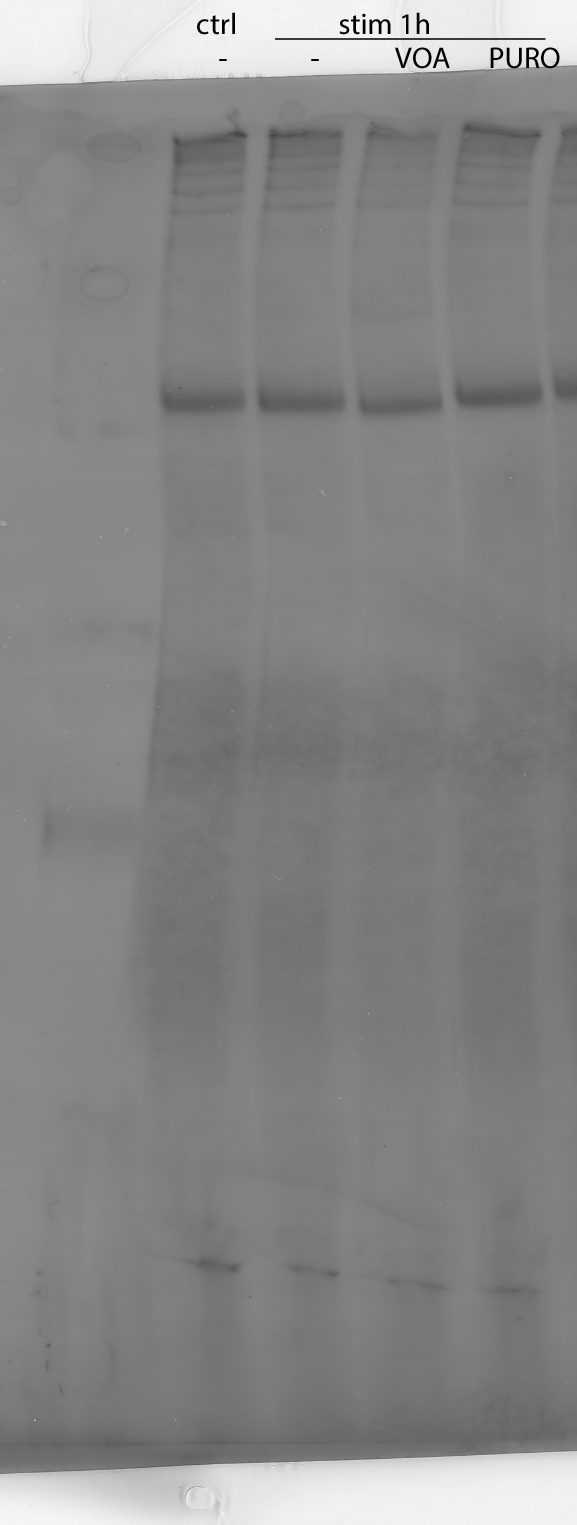

Supplement: Supplementary file 7 — Source Data for Figure 4 [file EMBR-21-e48882-s005.zip › Figure4_Source_Data/EMBOR-2019-48882V3-Figure_4C_Source_Data-sd(1).tif]

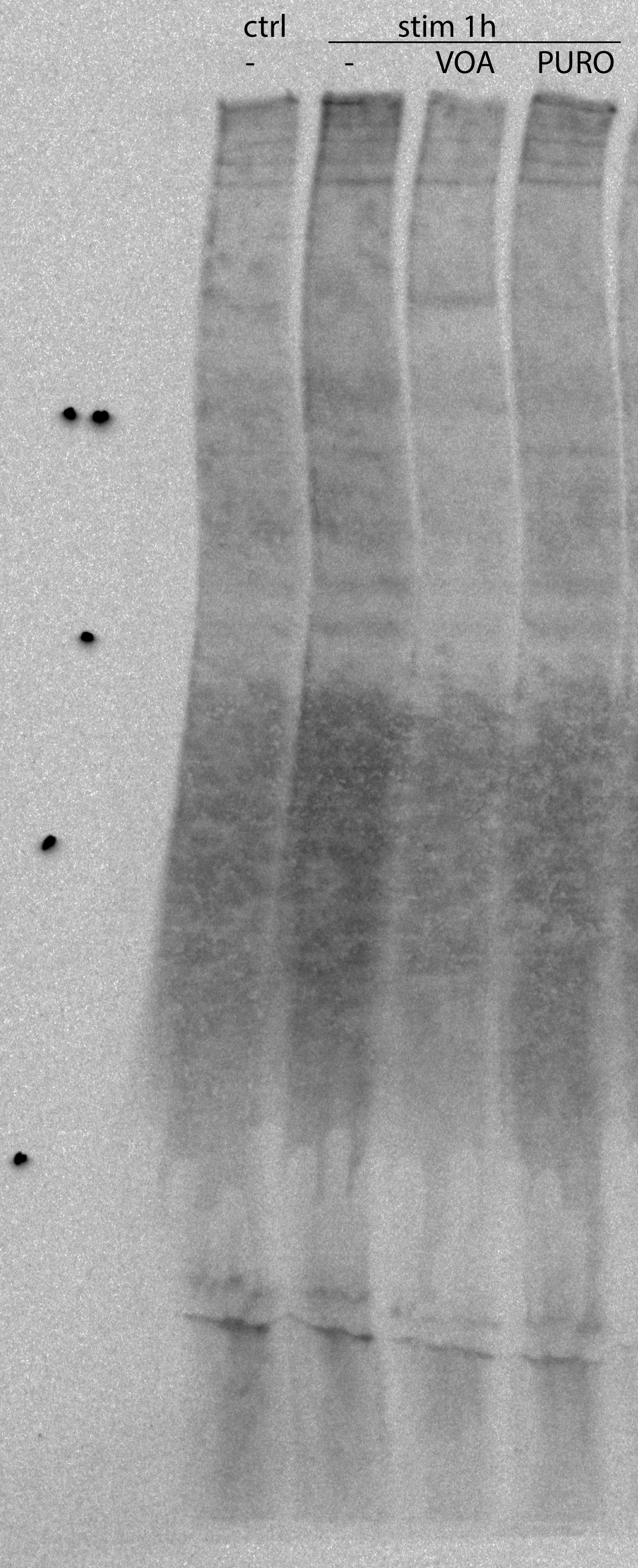

Supplement: Supplementary file 7 — Source Data for Figure 4 [file EMBR-21-e48882-s005.zip › Figure4_Source_Data/EMBOR-2019-48882V3-Figure_4C_Source_Data-sd.tif]

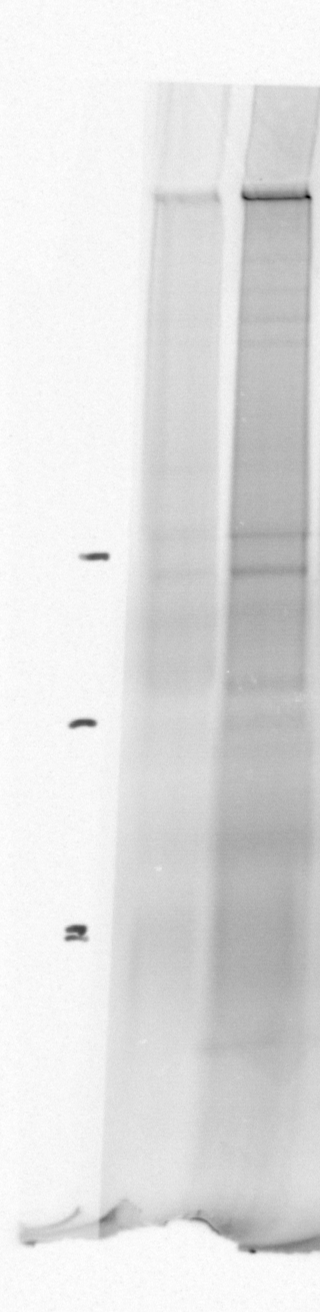

Supplement: Supplementary file 7 — Source Data for Figure 4 [file EMBR-21-e48882-s005.zip › Figure4_Source_Data/EMBOR-2019-48882V3-Figure_4A_Source_Data-sd(1).tif]
